# Effects of body mass index on relationship status, social contact and socio-economic position: Mendelian randomization and within-sibling study in UK Biobank

**DOI:** 10.1093/ije/dyz240

**Published:** 2019-12-04

**Authors:** Laura D Howe, Roshni Kanayalal, Sean Harrison, Robin N Beaumont, Alisha R Davies, Timothy M Frayling, Neil M Davies, Amanda Hughes, Samuel E Jones, Franco Sassi, Andrew R Wood, Jessica Tyrrell

**Affiliations:** 1 MRC Integrative Epidemiology Unit at the University of Bristol, Population Health Sciences, Bristol Medical School, University of Bristol, Bristol, UK; 2 Genetics of Complex Traits, University of Exeter Medical School, RILD Level 3, Royal Devon & Exeter Hospital, Exeter, UK; 3 Research and Evaluation Division, Public Health Wales, 2 Capital Quarter, Cardiff, UK; 4 Centre for Health Economics and Policy Innovation, Imperial College Business School, London, UK

**Keywords:** Body mass index, obesity, socio-economic, cohabitation, social contact, siblings, Mendelian randomization

## Abstract

**Background:**

We assessed whether body mass index (BMI) affects social and socio-economic outcomes.

**Methods:**

We used Mendelian randomization (MR), non-linear MR and non-genetic and MR within-sibling analyses, to estimate relationships of BMI with six socio-economic and four social outcomes in 378 244 people of European ancestry in UK Biobank.

**Results:**

In MR of minimally related individuals, higher BMI was related to higher deprivation, lower income, fewer years of education, lower odds of degree-level education and skilled employment. Non-linear MR suggested both low (bottom decile, <22 kg/m^2^) and high (top seven deciles, >24.6 kg/m^2^) BMI, increased deprivation and reduced income. Non-genetic within-sibling analysis supported an effect of BMI on socio-economic position (SEP); precision in within-sibling MR was too low to draw inference about effects of BMI on SEP. There was some evidence of pleiotropy, with MR Egger suggesting limited effects of BMI on deprivation, although precision of these estimates is also low. Non-linear MR suggested that low BMI (bottom three deciles, <23.5 kg/m^2^) reduces the odds of cohabiting with a partner or spouse in men, whereas high BMI (top two deciles, >30.7 kg/m^2^) reduces the odds of cohabitation in women. Both non-genetic and MR within-sibling analyses supported this sex-specific effect of BMI on cohabitation. In men only, higher BMI was related to lower participation in leisure and social activities. There was little evidence that BMI affects visits from friends and family or having someone to confide in.

**Conclusions:**

BMI may affect social and socio-economic outcomes, with both high and low BMI being detrimental for SEP, although larger within-family MR studies may help to test the robustness of MR results in unrelated individuals. Triangulation of evidence across MR and within-family analyses supports evidence of a sex-specific effect of BMI on cohabitation.

**Key Messages**

Studies have demonstrated stigma and discrimination against people who are overweight or obese in social, educational and employment settings.
Using Mendelian randomization (MR), a technique that uses genetic data to overcome confounding and reverse causality, we found evidence of sex-specific effects of body mass index (BMI) on the likelihood of being in a cohabiting relationship with a partner or spouse: in men, lower BMI was associated with being less likely to live with a partner or spouse, whereas in women, higher BMI was associated with being less likely to live with a partner or spouse. These results were robust to the use of both non-genetic and within-family MR analyses, which address potential biases in the main MR analysis due to family-level effects. Higher BMI was associated with lower participation in leisure activities in men but not women in MR of unrelated individuals; effects of BMI on other measures of social contact were not observed.
In our main MR analysis, we found evidence of effects of BMI on several domains of socio-economic position (income, deprivation, education, skilled employment), with BMIs at both ends of the distribution (both high and low BMI) leading to lower income and higher levels of deprivation. Findings were similar in non-genetic within-sibling analyses. However, in within-family MR analyses, confidence intervals were extremely wide, making inferences from these analyses challenging.

## Introduction

Lower socio-economic position (SEP) is associated with higher body mass index (BMI) and greater risk of obesity in high-income countries.^1–4^ People with higher BMI are more likely to experience weight-related stigma or discrimination, lower self-esteem, and physical and mental ill-health,^5–13^ all of which could potentially affect social, educational and employment outcomes, meaning that the relationships between social factors and BMI could be bidirectional. Currently, actions to address the increasing prevalence of obesity are supported by strong links between BMI and health outcomes, but demonstrating effects of BMI on social and socio-economic outcomes could augment the impetus for policy makers across sectors to act to prevent obesity, with the potential for greater societal benefits.

A key challenge to studying the downstream social and socio-economic consequences of BMI is reverse causality and confounding by earlier life factors such as parental SEP. Natural experiments are therefore needed. Siblings share their family environment and therefore if siblings discordant for obesity also differ with respect to SEP, this suggests the association is not due to confounding by shared family-level factors. In a study of male Swedish siblings, men who were obese as teenagers (*n* = 2600, of whom 95% had a sibling who was not obese) had an income 9% lower than men who were not obese as teenagers.^14^

Mendelian randomization (MR) is an alternative approach that uses genetic variants related to an exposure of interest (here, BMI) as instrumental variables.^15^ The approach exploits the natural experiment of genetic variants being randomly assigned at conception, which means they are less likely to be associated with factors that would confound a traditional analysis and should not suffer from reverse causality. A previous MR study in 120 000 UK Biobank participants provided evidence of an effect of higher BMI on several socio-economic outcomes, particularly lower income and higher area-level deprivation in women.^16^ Using data from over 350 000 participants in UK Biobank, we build on this by extending the analysis to additionally examine effects of BMI on important social outcomes—cohabiting relationship status and three measures of social contact. Given evidence that both low BMI and high BMI are associated with adverse outcomes,^14^^,^^17–19^ we also use novel MR methods to assess non-linearities in the effects of BMI on social and socio-economic outcomes. Recent evidence suggests that family-level effects (dynastic effects and assortative mating) can confound MR analyses.^20^ To interrogate whether any effects of BMI on social and socio-economic outcomes are robust to family-level effects, we therefore additionally conduct non-genetic and MR analyses within samples of siblings.

## Methods

UK Biobank is a study of over 500 000 individuals aged between 37 and 73 years recruited between 2006 and 2010.^21^ UK Biobank received ethics approval from the National Health Service National Research Ethics Service (ref 11/NW/0382). The data are available via application directly to UK Biobank. Exposure and outcome measures were defined from the baseline assessment centre. BMI was calculated from measured weight (kg)/height (m)^2^ and transformed to a normal distribution using the inverse normal function.

We used six measures of SEP (see Supplementary material, available as Supplementary data at *IJE* online):

Townsend deprivation index (TDI); an area-based measure; higher scores indicate higher deprivation
Annual household income
Job class; skilled versus unskilled
Employment status; employed (or self-employed) versus unemployed
Years in education
Degree status; degree-level education or lower

Note that some of these aspects of SEP may be expected to affect one another; for example, education can affect employment, income and area of residence (i.e. deprivation level).

Participants reporting that they lived with a ‘husband, wife or partner’ were defined as being in a cohabiting relationship.

We used three measures of social contact (see Supplementary material, available as Supplementary data at *IJE* online):

Visits from friends and family: less than weekly versus weekly or more visits
Participation in leisure and social activity: no activity versus any
Confiding in others: less than weekly versus weekly or more

Our main analysis is restricted to unrelated participants of white European ancestry, defined by Principal Component Analysis of genetic data (see Supplementary material, available as Supplementary data at *IJE* online). Participants were excluded if they had missing data for BMI (*n* = 1494) or a given outcome (Table 1). Within-family analyses are further restricted to families with at least two siblings.

**Table 1.** Characteristics of the 378 244 participants of European ancestry with valid genetic, body mass index and at least one outcome measure; NA, not applicable

Characteristic	All (*n* = 378 244)	Men (*n* = 174 358)	Women (*n* = 203 886)	*P* value^a^
Mean age at recruitment in years (SD)	57.2 (8.0)	57.5 (8.1)	57.0 (7.9)	<1 × 10^−15^
No. male sex (%)	174 358 (46.1%)	NA	NA	NA
Mean body mass index in kg/m^2^ (SD)	27.4 (4.8)	27.8 (4.2)	27.0 (5.2)	<1 × 10^−15^
Socio-economic position measures				
Mean Townsend Deprivation Index^b^ (SD)	−1.48 (2.99)	−1.44 (3.05)	−1.51 (2.94)	<1 × 10^−15^
Annual household income, *n* (%)				
<£18 000	70 841 (18.7)	30 536 (17.5)	40 305 (19.8)	<1 × 10^−15^
£18 000–£30 999	82 818 (21.9)	38 224 (21.9)	44 594 (21.9)	
£31 000–£51 999	86 024 (22.7)	42 443 (24.4)	43 581 (21.4)	
£52 000–£100 000	68 240 (18.0)	35 598 (20.4)	32 642 (16.0)	
>£100 000	18 194 (4.8)	9720 (5.6)	8474 (4.2)	
Mean number of years spent in education (SD)	15.0 (5.1)	15.3 (5.1)	14.6 (5.1)	<1 × 10^−15^
Number with a degree, *n* (%)	179 444 ( 47.4)	83 869 (48.1)	95 575 (46.9)	<1 × 10^−15^
Number with a skilled job, *n* (%)^c^	199 992 (81.6)	97 908 (83.1)	102 084 (80.2)	<1 × 10^−15^
Number of participants in employment, *n* (%)	216 248 (57.2)	104 979 (60.2)	111 269 (54.6)	<1 × 10^−15^
Social contact measures				
Cohabit with a partner or spouse, *n* (%)	277 399 (73.3)	134 062 (76.9)	143 337 (70.3)	<1 × 10^−15^
At least weekly visits from friends or family, *n* (%)	294 864 (78.0)	127 627 (73.2)	167 237 (82.0)	<1 × 10^−15^
Weekly participation in leisure and social activity, *n* (%)	262 868 (69.5)	121 596 (69.7)	141 272 (69.3)	0.031
At least weekly opportunities to confide in someone, *n* (%)	274 092 (72.5)	119 322 (68.4)	154 770 (75.9)	<1 × 10^−15^

a *P* values were determined in age- and sex-adjusted linear and logistic regression models.

b Higher values of the Townsend Deprivation Index imply higher levels of deprivation, i.e. lower socio-economic position.

c Percentage given as those from those reporting a job class.

## Statistical analysis

For TDI, years in education and income, we converted the data to a normal distribution using the inverse normal distribution function and report standard deviation (SD) effect sizes. Analyses using these normalized variables were then adjusted for age, sex, assessment centre and five (within-European) ancestry principal components. To convert results back to meaningful units after analysis, we multiplied our SD βs by a 1 SD change in the socio-economic status measure. For example, a 1 SD change in TDI was equivalent to 2.99 units. Therefore, a 0.05 SD change equated to a 0.150 unit change in deprivation. The betas represent the SD change in the outcome per SD change in BMI. The SD change in BMI equates to ∼4.8 kg/m^2^. Analyses using the original variables within ordinal logistic regression models yielded the same pattern of results and are therefore not described further.

### Linear and logistic regression

We regressed each outcome against BMI using linear or logistic regression, with age and sex as covariates. Data on factors that could potentially confound associations (e.g. parental SEP) are very limited in UK Biobank. As a sensitivity analysis, we adjusted for maternal smoking and birth weight (both reported by the participants at the baseline assessment visit), potential proxies for early life factors that may confound the observational associations.

### Genetic risk score for BMI

Genetic variants for BMI were selected from UK Biobank’s imputation dataset.^22^ We selected 73 of 76 variants associated with BMI at genome-wide significance in all people of European ancestry in the Genetic Investigation of ANthropometric Traits (GIANT) consortium studies (Supplementary Table S1, available as Supplementary data at *IJE* online).^23^ Three variants were excluded because they were known to have pleiotropic effects on other traits [rs11030104 (*BDNF* reward phenotypes including smoking), rs13107325 (*SLC39A8* lipids, blood pressure), rs3888190 (*SH2B1* multiple traits)].^16^^,^^23^

The 73 variants were combined into a genetic risk score (GRS). Each variant was weighted by its effect size (β-coefficient) from the primary GWAS that did not include any UK Biobank data (equation 1).^23^ The weighted score was then rescaled to reflect the number of trait-raising alleles (equation 2).

(1) $$\mathrm{Weighted}\;\mathrm{score}=\beta_{1}\;x\;{\mathrm{SNP}}_{1}+\;\beta_{2}\;x\;{\mathrm{SNP}}_{2\;}+\cdots \beta_{n}\;x\;{\mathrm{SNP}}_{n}$$

 

(2) $$\mathrm{Weighted}\;\mathrm{genetic}\;\mathrm{risk}\;\mathrm{score}=\frac{\mathrm{weighted}\;\mathrm{score}\;x\;\mathrm{number}\;\mathrm{of}\;\mathrm{SNPs}}{\mathrm{sum}\;\mathrm{of}\;\mathrm{the}\;\beta \;\mathrm{coefficients}}$$

### Mendelian randomization in minimally related individuals

We employed the two-stage-least-squares regression estimator that uses predicted levels of BMI per genotype and regresses the outcome against these predicted values. For binary outcomes, the analysis was done in two stages. Firstly, the association between the BMI GRS and BMI was assessed. The predicted values from this regression were used as the independent variable and the binary or ordinal outcomes as the dependent variable in logistic regression models. Robust standard errors were used. For continuous outcomes we used the ivreg2 command in Stata.

### Differences between men and women

To test whether associations differ in men and women, we repeated linear and logistic regression and MR analyses separately in each sex. The selected BMI genetic variants have similar effects in men and women and therefore the same variants and GRS were used in sex-stratified analyses. Beta values for men and women were compared using Fisher’s *z*-score method (equation 3).^24^
 

(3) $$z=\frac{{\mathrm{Beta}}_{\mathrm{male}}-{\mathrm{Beta}}_{\mathrm{female}}}{\sqrt{{\mathrm{SE}}_{\mathrm{male}}^{2}+{\mathrm{SE}}_{\mathrm{female}}^{2}}}$$

### Sensitivity analyses

#### Mechanisms underlying associations with cohabitation

Any associations between BMI and cohabitation with a partner/spouse could result from (1) associations between BMI and partnership formation, (2) associations between BMI and separation/divorce, (3) mortality of partners (assortative mating could result in people of high BMI being more likely to have partners who also have high BMI, and hence who are more likely to die at a younger age). To help unpick these mechanisms, we examined the associations of BMI with separation from partner/spouse in the past 2 years and death of a partner or spouse in the past 2 years. Due to low numbers of people reporting these two events (7745 reported separations, 4415 reported partner deaths), these analyses were limited to logistic regression. We also repeated our main logistic regression and MR analyses for cohabitation stratifying by median age, <58 years or ≥58 years, with the hypothesis that if partner death was contributing to the overall associations, the association would be stronger in the older age group.

#### Effect of household size on associations with income

We explored the relationships between BMI and annual household income per capita by adjusting the income variable for the number of people living in the household.

#### Effect of ill-health

We repeated our analyses in a subset of 88 323 individuals with no known health problems to investigate whether associations were independent of poor health.

#### Evaluating pleiotropy

A potential source of residual confounding in MR studies is horizontal pleiotropy.^25^ A range of two-sample MR analyses were performed to assess the validity of the MR assumptions and investigate pleiotropy.^25^ Full details of the methods used^26^^,^^27^ are in the Supplementary material, available as Supplementary data at *IJE* online.

#### Associations with potential confounders

We explored associations of BMI and the BMI GRS with maternal smoking and birth weight.

#### Using a larger BMI genetic risk score

Recently, further genetic variants for BMI have been identified using ∼700 000 individuals of European ancestry,^28^ including individuals from the UK Biobank. These newly discovered 941 variants explain more variation in BMI (6% compared with <3% for the GIANT study^23^). We did not use a GRS based on this study as our main analysis because they were discovered using the UK Biobank and therefore may bias our findings towards the observational estimate.^29^ However, as a sensitivity analysis, we repeat our MR analysis using this GRS.

### Analysis of non-linearity

To explore non-linear relationships, i.e. whether the change in outcomes is not constant across each unit increase in BMI, we employed non-linear MR. This approach assessed how the association of BMI with each outcome differs across deciles of IV-free BMI (residuals from a regression of BMI on the genetic instrumental variable; herein referred to as ‘BMI deciles’ for brevity). Deciles were chosen a priori to achieve a reasonable balance between statistical power and granularity. This analysis was carried out using the nlmr package (https://github.com/jrs95/nlmr) in R.^30^ Full details are available as Supplementary data at *IJE* online.

### Within-family analysis

To assess whether any effects of BMI are robust to family-level effects, we carried out non-genetic and MR analysis within siblings, centring BMI (for non-genetic analysis) or the BMI GRS (for MR analysis) within each family; further details are in the Supplementary material, available as Supplementary data at *IJE* online and Brumpton *et al*.^20^ Analyses were conducted for both sexes combined and a sex-stratified analysis in families with two or more male/female siblings in the study. Due to power concerns, within-family analyses are limited to models that assume a linear effect of BMI.

## Results

BMI was available for 378 244 unrelated individuals of white European ancestry with at least one of the outcome measures (Table 1). The 73 single nucleotide polymorphism (SNP) GRS explained 1.7% of the variance in BMI; the 941 SNP GRS explained 5.0% of the variation.

### Association of BMI with SEP

**Figure 1.**
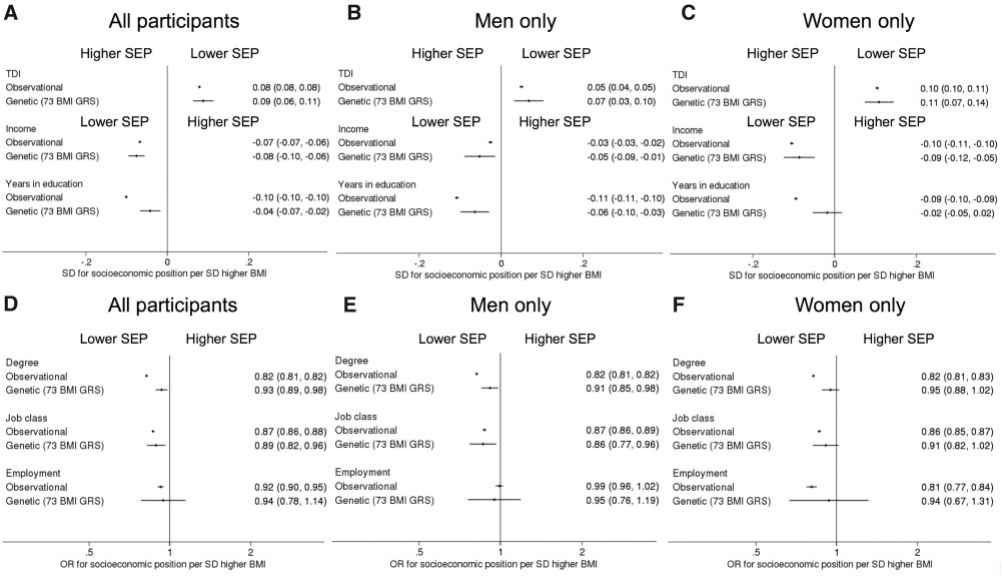
Forest plots of the linear and logistic regression and MR associations between a 1 SD higher body mass index (BMI) and six measures of socio-economic position (SEP). (A–C) represent the differences in continuous SEP measures (TDI, income and years in education) in (A) all individuals, (B) men only and (C) women only. (D–F) represent differences in binary SEP measures (degree, skilled job and employment status) in (D) all individuals, (E) men only and (F) women only. Higher and lower SEP are marked on the plots as higher SEP and lower SEP. Note TDI is the opposite way round to the other SEP measures, with higher values representing more deprivation.

In linear and logistic regression models, higher BMI was associated with higher deprivation, lower income, fewer years in education and lower odds of holding a degree, being employed and working in a skilled profession (Figure 1A–F; Supplementary Table S2, available as Supplementary data at *IJE* online). Adjustment for maternal smoking and birth weight did not alter results (Supplementary Table S3, available as Supplementary data at *IJE* online).

MR estimates suggested that higher BMI reduces SEP (Figure 1A–F; Supplementary Table S2, available as Supplementary data at *IJE* online); a 1 SD higher BMI (4.8 kg/m^2^ in UK Biobank) (1) increased deprivation by 0.09 SD [95% confidence interval (CI): 0.06, 0.11, *P* = 4 × 10^−^^12^], which equates to approximately one-third of a decile, (2) decreased income by 0.07 SD (95%CI: 0.04, 0.10, *P* = 4 × 10^−^^7^) which approximates to £1660 less income per annum (95%CI: £950, £2380), (3) decreased years of education by 0.04 SD (95%CI: 0.02, 0.07, *P* = 0.001), which equates to ∼3 months, (4) decreased the likelihood of having a degree by 7% [odds ratio (OR) 0.93, 95%CI: 0.89, 0.98, *P* = 0.007] and (5) decreased the likelihood of having a skilled job by 11% (OR 0.89, 95%CI: 0.82, 0.96, p = 0.003). There was little evidence of an effect of BMI on being employed: OR = 0.94, 95% CI 0.78, 1.14, *P* = 0.6. Statistical tests for sex differences provided little evidence of associations differing between men and women (Supplementary Table S2, available as Supplementary data at *IJE* online).

### Association of BMI with relationship status

**Figure 2.**
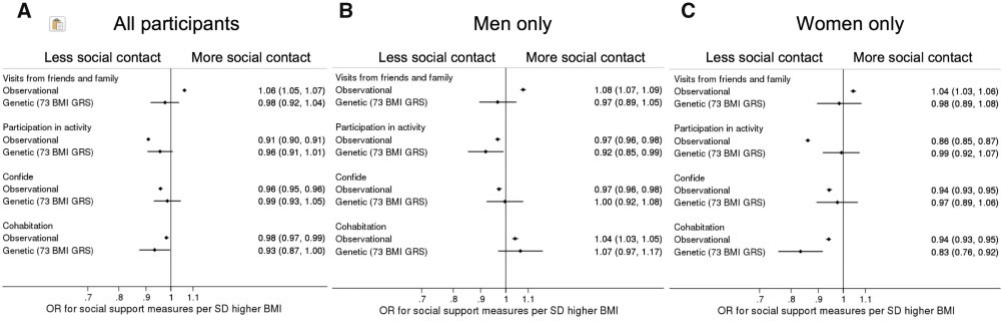
Forest plots of the linear and logistic regression and Mendelian randomization associations between a 1 SD higher BMI and the four social contact measures in (A) all individuals, (B) men only and (C) women only. Higher and lower social contact is marked on the plots as more social contact and less social contact.

In logistic regression analyses, higher BMI was associated with higher odds of cohabitation with a partner or spouse in men, and lower odds of cohabitation with a partner or spouse in women (Figure 2; Supplementary Table S2, available as Supplementary data at *IJE* online). MR analysis provided evidence that higher BMI reduces the odds of cohabitation with a partner or spouse in women (OR: 0.83, 95%CI: 0.76, 0.92, *P* = 0.0002). In men, there was little evidence from MR analyses for an association between BMI and cohabiting with a spouse/partner (OR: 1.07, 95% CI: 0.97, 1.17, *P* = 0.2). There was strong evidence for sex differences in the MR results, *P* = 0.0004.

### Association of BMI with social contact

In logistic regression analyses, higher BMI was associated with higher odds of seeing friends and family on a weekly basis, lower odds of having someone to confide in regularly and lower odds of participating in leisure activities (Figure 2, Supplementary Table S2, available as Supplementary data at *IJE* online). There was little difference in results with adjustment for maternal smoking and birth weight (Supplementary Table S3, available as Supplementary data at *IJE* online). In MR analyses, higher BMI decreased the likelihood of participation in leisure and social activities in men (OR: 0.92, 95% CI: 0.85, 0.99, *P* = 0.03) but not women (OR: 0.99, 95% CI: 0.92, 1.07, *P* = 0.9), although evidence for sex differences was not strong (*P* = 0.2). There was no evidence for an effect of BMI on the odds of weekly visits from friends and family or having someone to regularly confide in.

### Sensitivity analyses

#### Mechanisms underlying associations with cohabitation

Logistic regression analyses in men and women combined demonstrated that higher BMI was associated with lower odds of divorce/separation in the past 2 years (Supplementary Table S4, available as Supplementary data at *IJE* online). Higher BMI was associated with higher chance of a partner or spouse having died in the last 2 years (OR 1.05, 95% CI 1.02 to 1.07, *P* = 0.001). In age-stratified analyses (Supplementary Table S5, available as Supplementary data at *IJE* online), MR results indicate a strong association between higher BMI and lower chance of being in a cohabiting relationship in people <58 years old, which is limited to women (OR in women 0.74, 95% CI 0.64 to 0.85, *P* = 1 × 10^−^^5^; OR in men 1.07, 95% CI 0.94 to 1.21, *P* = 0.3). These associations were not seen in people ≥58 years old, although CIs overlapped with those for people aged <58 years (OR in women ≥58 years 0.93, 95% CI 0.81–1.06, *P* = 0.3; OR in men ≥58 years 1.08, 95% CI 0.94–1.24, *P* = 0.3].

#### Effect of household size on associations with income

Accounting for the number of individuals in the household did not meaningfully alter the relationship between BMI and income (Supplementary Table S6, available as Supplementary data at *IJE* online).

#### Effect of ill-health

In a subset of individuals who reported no health problems, the linear and logistic regression results were generally consistent with the main analyses. MR analyses were mostly in the same direction as the main analysis, but coefficients were closer to the null and CIs were wide; there was only strong evidence for an effect of higher BMI on higher deprivation in all individuals and women only (Supplementary Table S7, available as Supplementary data at *IJE* online).

#### Evaluating pleiotropy

There was some evidence of pleiotropy when years spent in education or having a degree were the outcome variable when using Egger-MR, but not for other outcomes. Some differences in results were seen between the main MR analysis and MR Egger. For example, MR Egger found little strong evidence of an effect of BMI on deprivation and income, and suggested a small positive effect on education (Supplementary Table S2, available as Supplementary data at *IJE* online).

#### Associations with confounders

Observationally, maternal smoking is associated with a 0.16 (95% CI: 0.15, 0.16) SD higher BMI and a 1 SD higher birthweight is associated with 0.03 (95%CI: 0.03, 0.04) SD higher BMI. A genetically instrumented 1 SD higher BMI was associated with a 0.075 SD higher birthweight (95% CI: 0.040, 0.112) and 1.17 higher odds of maternal smoking (95% CI: 1.11, 1.25).

#### Using a larger BMI genetic risk score

Using the 941 BMI variant GRS in many cases suggested stronger effects of BMI than the main analysis (Supplementary Table S2, available as Supplementary data at *IJE* online).

### Non-linear Mendelian randomization

**Figure 3.**
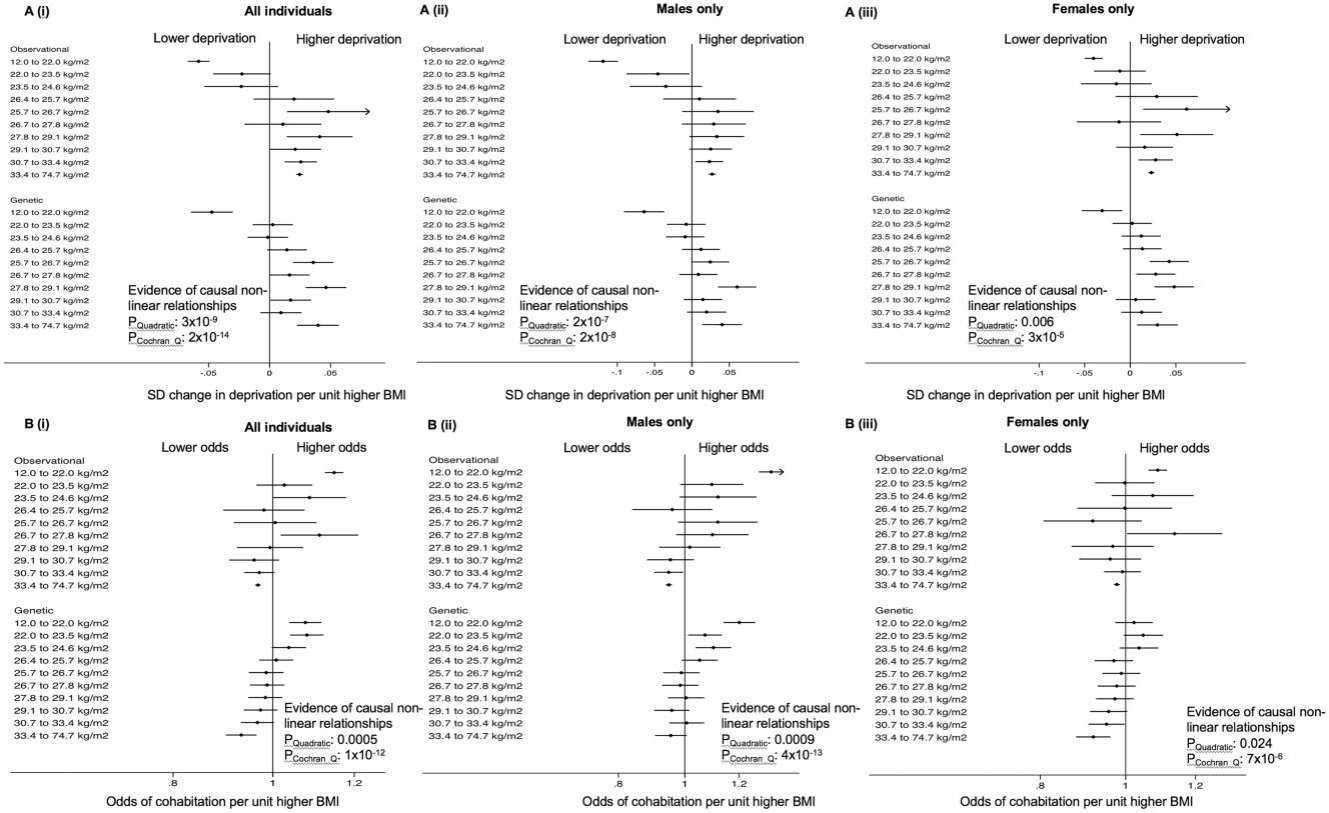
Dot plot exploring the non-linear associations of BMI with TDI and cohabitation, in deciles of genetically-determined BMI using the piecewise linear model from non-linear MR analysis. The *P*-values presented for causal non-linear relationships are from the piecewise linear model (*P*_Quadratic_ and *P*_Cochran Q_). Full results of non-linear analyses are provided in Supplementary Tables S8 and S9, available as Supplementary data at *IJE* online.

There was evidence that both low and high BMI resulted in higher deprivation (Figure 3). For people in the lowest BMI decile (<22 kg/m^2^), higher BMI was associated with lower deprivation. Detrimental effects of high BMI on deprivation were evident for the top seven BMI deciles (>24.6 kg/m^2^). Both low and high BMI were also associated with lower income (Supplementary Figure S1, available as Supplementary data at *IJE* online).

Sex differences were apparent in the non-linear MR results for cohabitation with a partner or spouse (Figure 3). In women, the results suggested an association between higher BMI and lower odds of cohabitation with a partner or spouse. This association was strong for the top two BMI deciles (>30.7 kg/m^2^). In contrast, in men there was evidence that low BMI (bottom three deciles, <24.6 kg/m^2^) was associated with reduced odds of cohabitation with a partner or spouse.

For other outcomes, MR analyses did not provide evidence of non-linear relationships. Full results are in Supplementary Tables S8 and S9, available as Supplementary data at *IJE* online.

### Within-sibling analysis

**Figure 4.**
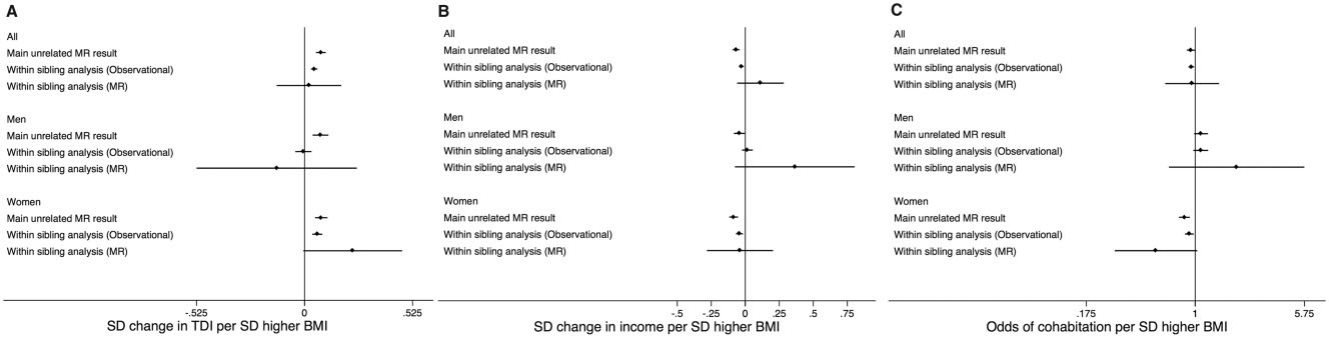
Forest plots of the results from MR of unrelated participants and non-genetic and MR within-sibling analyses for the associations of BMI with (A) deprivation, (B) income and (C) cohabitation. Full results of within-sibling analyses are provided in Supplementary Table S10, available as Supplementary data at *IJE* online.

There were up to 39 865 siblings within 19 475 families (Supplementary Table S10, available as Supplementary data at *IJE* online; Figure 4). For cohabitation with a partner or spouse, within-sibling analyses provided evidence that was consistent with our main analysis. Both non-genetic and MR analysis within siblings supported a relationship of higher BMI being associated with lower odds of cohabitation in women and higher odds of cohabitation in men, although CIs spanned the null for non-genetic sibling analyses in men and for within-sibling MR in women. For socio-economic outcomes, non-genetic within-sibling analysis tended to support the main analysis results. For all outcomes other than cohabitation, CIs for within-sibling MR were extremely wide, and consistent with both the null and with the estimates from MR analysis in unrelated individuals.

## Discussion

Ill-health, stigma and discrimination experienced by people with BMIs at either end of the distribution could result in adverse social and socio-economic consequences. Using triangulation across a range of methods, we have explored the social and socio-economic consequences of BMI. MR analysis in ∼350 000 unrelated participants suggested that both low BMI (<22 kg/m^2^) and high BMI (>24.6 kg/m^2^) may lead to lower SEP in both men and women, that high BMI is related to lower chance of being in a cohabitating relationship for women but lower BMI is related to lower chance of being in a cohabiting relationship for men, and that higher BMI can lead to men being less likely to participate in leisure activities. We found little evidence of a causal effect of BMI on contact with friends and family or having someone to confide in. Non-genetic analyses using data from samples of siblings tended to support the conclusions of our main MR analyses that high BMI leads to lower SEP. These non-genetic sibling analyses control for family-level effects that may be a source of bias in the main MR analysis, but can still be subject to confounding by individual-level factors. Within-sibling MR analysis, which should be robust to both family- and individual-level confounding, had precision that was too low to reach firm inference for SEP outcomes. However, for cohabitation, we have shown robust evidence across three methods (MR in unrelated individuals and non-genetic and MR within-sibling models) that in women, higher BMI reduces the odds of cohabitation with a partner or spouse, whereas the opposite occurs in men.

We observe strong sex differences in the effects of BMI on odds of being in a cohabiting relationship. In men, there was evidence of low BMI being associated with reduced odds of cohabitation with a partner or spouse. In contrast, for women there was a strong effect of higher BMI on lower odds of cohabitation. These effects were seen in the main MR analysis, in non-linear MR, and in both non-genetic and MR analysis exploiting within-sibling differences in BMI/BMI GRS. The sex differences we observe may be indicative of gender differences in the cultural idealization and social values for body size, with thinness being culturally valued in women, but perceived strength being valued in men.^31^ Associations of BMI with cohabitating relationship status could arise due to effects of BMI on partnership formation, divorce/separation, and/or partner death (if partnerships are more likely to form within couples of similar BMI). We were unable to assess the role of partnership formation with UK Biobank, but our results suggest that divorce/separation in the past 2 years (we could not evaluate the role of earlier separations) and partner death are unlikely to fully explain the associations we observe.

We hypothesized that associations between high BMI and lower SEP could arise due to the mental and physical health consequences of high BMI, and/or via social mechanisms including cultural norms and expectations of body size and related stigma and discrimination.^7^ Although MR analysis is less likely to be confounded than standard observational analyses, family-level confounding through mechanisms such as assortative mating and dynastic effects is still possible.^20^ Comparing siblings is one way to avoid such biases, as the family-environment is similar for children growing up in the same household. Non-genetic within-sibling analyses should be robust to family-level confounding, but may still be affected by bias due to individual-level confounding. In contrast, within-sibling MR should be robust to both family- and individual-level confounding. For SEP outcomes, non-genetic analysis within samples of siblings tended to support the conclusions of our main MR analysis, suggesting that high BMI is detrimental for SEP. Within-sibling MR analyses for SEP outcomes had very low precision; all CIs were extremely wide. Thus, no firm conclusions can be reached from these analyses. In a previous analysis of UK Biobank and a Norwegian study combined, which approximately doubles the number of siblings included in the analysis, within-family MR found little evidence of an effect of BMI on educational attainment, but precision was still low to exclude the possibility that BMI did not affect educational attainment.^20^ The effects of BMI on SEP seen in our main MR analysis and non-genetic within-sibling analysis were also observed in a previous, much larger, sibling study, which suggested effects of BMI on education, income, cognitive and non-cognitive skills.^14^ We observed a similar pattern of associations in MR analysis of unrelated participants in the subset of participants reporting no health conditions, suggesting that ill-health is not the only mechanism driving any effects of BMI. However, this analysis was limited by small numbers of people reporting no health conditions, and by the limitations of the self-reported and binary measures of health within this study.

The adverse effects of low BMI suggested by our main MR analysis were of similar magnitude to the adverse effects of high BMI, mirroring the results of a previous sibling-comparison study.^14^ However, the effects of high BMI were seen across a wider range of BMIs and a larger number of people, and so, if real, will have greater total societal implications. In non-linear MR, the effects of low BMI on higher deprivation and lower income were observed in the bottom decile of BMI <22 kg/m^2^. The majority of people in this group have a BMI within the ‘recommended’ range (18.5–24.9 kg/m^2^). For the effect of low BMI on lower odds of cohabitation in men, this association is apparent for BMIs below 24.6 kg/m^2^, i.e. encompassing almost the entire range of ‘recommended’ BMI. Only 2000 individuals in UK Biobank are underweight (BMI < 18.5 kg/m^2^), meaning that there is insufficient power to apply any MR or within-sibling analyses to this subgroup.

Several of our results from MR analyses differ considerably from our analysis using linear/logistic regression. Notably, associations of BMI with educational outcomes are far weaker when analysed using MR, and associations with social contact seen in logistic regression analyses were null in MR. This suggests that the linear and logistic regression results are strongly biased from reverse causality or confounding. Reverse causality is particularly problematic for educational attainment, since we are looking at BMI measured in mid-life. There is very little data in UK Biobank on factors that could confound the associates (e.g. parental SEP), so evaluating the presence and extent of confounding is challenging. Adjustment for maternal smoking and birth weight, potential proxies for early life confounders, did not alter observational associations between BMI and SEP, despite associations of both factors with BMI. However, the BMI GRS was also associated with both birth weight and maternal smoking. While the association with birth weight is expected, the association with maternal smoking is less intuitive, but may reflect known causal relationships between BMI and smoking in the mothers.^32^

One of the key assumptions of MR is that the genetic variants used as an instrumental variable affect the outcome only through their effect on the exposure, i.e. the absence of horizontal pleiotropy.^25^ Our sensitivity analyses using two-sample MR approaches provided results that in some cases differed from the main analyses: for example, MR Egger suggested weak or no effect of BMI on deprivation and income, and a small positive effect on education. For education, MR Egger suggested the presence of pleiotropy, which may explain the difference between these results. For the other outcomes, the differences may be attributable to lower power in MR Egger analysis compared with the main analysis. When using an expanded BMI GRS, estimated effects of BMI tended to be of greater magnitude. However, this finding should be interpreted with caution, as the sample overlap between the identification of genetic variants and the participants in our analysis could have biased the results towards the observational associations.^29^ The association of the BMI GRS with BMI changes across the life course; the MR estimates reflect the average effect of BMI across the entire life-course.^33^

UK Biobank is restricted to participants born between 1938 and 1971; the social and socio-economic consequences of BMI may differ in younger generations who have grown up during the obesity epidemic. Furthermore, UK Biobank had only a 5% response rate.^21^^,^^34^ It is a relatively homogenous population, and our analyses were restricted to people of White European descent; generalizability of our findings may therefore be limited. Analysis has uncovered geographical patterning of both phenotypes and genotypes in UK Biobank, and shown that this structure can bias associations.^35^ The patterns of selection into studies such as UK Biobank can induce collider bias, with even modest influences on selection potentially leading to biased estimates.^36^ Such biases can equally affect instrumental variable analyses.^37^ Statistical methods to estimate causal effects from non-representative samples are currently under-developed and are an area of priority activity, as are efforts to improve the representativeness of participants in large-scale research studies such as UK Biobank. However a recent MR study in the UK Household Longitudinal Study and the English Longitudinal Study of Ageing, both nationally representative samples, also supports negative effects of higher BMI on a range of SEP outcomes.^38^ This suggests the associations seen in UK Biobank do not just reflect selection bias.

In summary, MR analyses in 350 000 unrelated individuals suggest effects of both high and low BMI on SEP and odds of being in a cohabiting relationship, with important sex differences in the effects of BMI on cohabitation. MR methods that are more robust to pleiotropy provided weaker evidence for cohabitation, with CIs crossing the null, but limited evidence of horizontal pleiotropy was observed with MR-Egger. For cohabitation, within-sibling non-genetic and MR analyses support these conclusions. For SEP outcomes, non-genetic within-sibling analyses supported a role for higher BMI influencing lower SEP, but precision was too low in within-family MR analyses to draw inference about the relationship between BMI and SEP outcomes.

## Author contributions

L.D.H. and J.T. conceived the study. Statistical analyses were performed by J.T., R.K. and S.H. L.D.H., R.K. and J.T. wrote the first draft of the manuscript. All authors contributed to study design, interpretation of results and provided comments and critical revisions for intellectual content. L.D.H. and J.T. accept full responsibility for the overall content as guarantors. The corresponding author attests that all listed authors meet authorship criteria and that no others meeting the criteria have been omitted.

## Funding

This work was supported by a Career Development Award from the UK Medical Research Council (MR/M020894/1) to L.D.H. L.D.H., S.H., N.M.D. and A.H. work in a unit funded by the University of Bristol and the UK Medical Research Council (MC_UU_12013/1, MC_UU_12013/9, MC_UU_00011/1). This work is part of a project entitled ‘social and economic consequences of health: causal inference methods and longitudinal, intergenerational data’, which is part of the Health Foundation’s Efficiency Research Programme. The Health Foundation is an independent charity committed to bringing about better health and health care for people in the UK. The Economics and Social Research Council (ESRC) support N.M.D. via a Future Research Leaders grant [ES/N000757/1]. S.E.J. is funded by the Medical Research Council (grant: MR/M005070/1). The funders had no role in the study design; in the analysis and interpretation of data; in the writing of the report; and in the decision to submit the article for publication. The authors would like to acknowledge the use of the University of Exeter High-Performance Computing (HPC) facility in carrying out this work.

**Conflict of interest:** None declared.

## Supplementary Material

dyz240_Supplementary_Data
