# Supplementary material for: Effects of body mass index on relationship status, social contact and socio-economic position: Mendelian randomization and within-sibling study in UK Biobank
Source: Int J Epidemiol. 2019 Dec 4;49(4):1173–84. doi: 10.1093/ije/dyz240 (PMC7750981; doi:10.1093/ije/dyz240)
Supplement: dyz240_Supplementary_Data [file dyz240_supplementary_data.zip › dyz240-suppl_data/ije-2019-03-0363-File007.docx]

| **Supplementary table S1:** Summary of the genetic variants for BMI for the 73 BMI GRS | | | | |  |  |  |  |
| --- | --- | --- | --- | --- | --- | --- | --- | --- |
| **Trait** | **Genetic variant** | **Locus** | **Exclude from score** | **Reason for exclusion** | **Trait raising allele** | **Trait lowering allele** | **Imputation quality** | **Beta representing SD change in BMI from the primary GWAS*** |
| BMI | rs1000940 | *RABEP1* | No | NA | G | A | 0.998767 | 0.019 |
| BMI | rs10132280 | *STXBP6* | No | NA | C | A | 0.989184 | 0.023 |
| BMI | rs1016287 | *FLJ30838* | No | NA | T | C | 0.998017 | 0.023 |
| BMI | rs10182181 | *ADCY3* | No | NA | G | A | 0.99677 | 0.031 |
| BMI | rs10733682 | *LMX1B* | No | NA | A | G | 0.966835 | 0.017 |
| BMI | rs10938397 | *GNPDA2* | No | NA | G | A | 1 | 0.04 |
| BMI | rs10968576 | *LINGO2* | No | NA | G | A | 1 | 0.025 |
| BMI | rs11030104 | *BDNF* | Yes | BMI-raising allele also associated with regular smoking (which itself has a causal effect on BMI in opposite direction) | A | G | 0.99901 | 0.041 |
| BMI | rs11057405 | *CLIP1* | No | NA | G | A | 1 | 0.031 |
| BMI | rs11126666 | *KCNK3* | No | NA | A | G | 0.997088 | 0.021 |
| BMI | rs11165643 | *PTBP2* | No | NA | T | C | 0.998186 | 0.022 |
| BMI | rs11191560 | *NT5C2* | No | NA | C | T | 0.999813 | 0.031 |
| BMI | rs11583200 | *ELAVL4* | No | NA | C | T | 0.994046 | 0.018 |
| BMI | rs1167827 | *HIP1* | No | NA | G | A | 1 | 0.02 |
| BMI | rs11688816 | *EHBP1* | No | NA | G | A | 0.993737 | 0.017 |
| BMI | rs11727676 | *HHIP* | No | NA | T | C | 1 | 0.036 |
| BMI | rs11847697 | *PRKD1* | No | NA | T | C | 1 | 0.049 |
| BMI | rs12286929 | *CADM1* | No | NA | G | A | 0.997012 | 0.022 |
| BMI | rs12401738 | *FUBP1* | No | NA | A | G | 0.995778 | 0.021 |
| BMI | rs12429545 | *OLFM4* | No | NA | A | G | 0.982609 | 0.033 |
| BMI | rs12446632 | *GPRC5B* | No | NA | G | A | 0.999769 | 0.04 |
| BMI | rs12566985 | *FPGT-TNNI3K* | No | NA | G | A | 0.998568 | 0.024 |
| BMI | rs12885454 | *PRKD1* | No | NA | C | A | 0.998387 | 0.021 |
| BMI | rs12940622 | *RPTOR* | No | NA | G | A | 0.99906 | 0.018 |
| BMI | rs13021737 | *TMEM18* | No | NA | G | A | 0.99987 | 0.06 |
| BMI | rs13078960 | *CADM2* | No | NA | G | T | 0.994176 | 0.03 |
| BMI | rs13107325 | *SLC39A8* | Yes | Missense Ala/Thr polymorphism located in exon 7 of SLC39A8, which encodes a zinc transporter that also transports cadmium and manganese. It is also associated with BP and HDL levels, and presumably these and the BMI effect are secondary to the metal ion transport variation. | T | C | 1 | 0.048 |
| BMI | rs13191362 | *PARK2* | No | NA | A | G | 0.995431 | 0.028 |
| BMI | rs1516725 | *ETV5* | No | NA | C | T | 0.996107 | 0.045 |
| BMI | rs1528435 | *UBE2E3* | No | NA | T | C | 0.997813 | 0.018 |
| BMI | rs1558902 | *FTO* | No | NA | A | T | 0.999785 | 0.082 |
| BMI | rs16851483 | *RASA2* | No | NA | T | G | 0.999167 | 0.048 |
| BMI | rs16951275 | *MAP2K5* | No | NA | T | C | 0.999393 | 0.031 |
| BMI | rs17001654 | *SCARB2* | No | NA | G | C | 0.976347 | 0.031 |
| BMI | rs17024393 | *GNAT2* | No | NA | C | T | 0.99093 | 0.066 |
| BMI | rs17094222 | *HIF1AN* | No | NA | C | T | 0.992428 | 0.025 |
| BMI | rs17405819 | *HNF4G* | No | NA | T | C | 0.999855 | 0.022 |
| BMI | rs17724992 | *PGPEP1* | No | NA | A | G | 0.991823 | 0.019 |
| BMI | rs1808579 | *C18orf8* | No | NA | C | T | 0.998227 | 0.017 |
| BMI | rs1928295 | *TLR4* | No | NA | T | C | 0.999945 | 0.019 |
| BMI | rs2033529 | *TDRG1* | No | NA | G | A | 0.992365 | 0.019 |
| BMI | rs2033732 | *RALYL* | No | NA | C | T | 1 | 0.019 |
| BMI | rs205262 | *C6orf106* | No | NA | G | A | 0.998614 | 0.022 |
| BMI | rs2075650 | *TOMM40* | No | NA | A | G | 1 | 0.026 |
| BMI | rs2112347 | *POC5* | No | NA | T | G | 1 | 0.026 |
| BMI | rs2121279 | *LRP1B* | No | NA | T | C | 0.992193 | 0.025 |
| BMI | rs2176598 | *HSD17B12* | No | NA | T | C | 1 | 0.02 |
| BMI | rs2207139 | *TFAP2B* | No | NA | G | A | 0.9998 | 0.045 |
| BMI | rs2245368 | *PMS2L11* | No | NA | C | T | 1 | 0.032 |
| BMI | rs2287019 | *QPCTL* | No | NA | C | T | 0.985702 | 0.036 |
| BMI | rs2365389 | *FHIT* | No | NA | C | T | 0.994976 | 0.02 |
| BMI | rs2650492 | *SBK1* | No | NA | A | G | 0.989836 | 0.021 |
| BMI | rs2820292 | *NAV1* | No | NA | C | A | 1 | 0.02 |
| BMI | rs29941 | *KCTD15* | No | NA | G | A | 1 | 0.018 |
| BMI | rs3101336 | *NEGR1* | No | NA | C | T | 1 | 0.033 |
| BMI | rs3736485 | *DMXL2* | No | NA | A | G | 0.995344 | 0.018 |
| BMI | rs3810291 | *ZC3H4* | No | NA | A | G | 1 | 0.028 |
| BMI | rs3817334 | *MTCH2* | No | NA | T | C | 1 | 0.026 |
| BMI | rs3849570 | *GBE1* | No | NA | A | C | 0.999768 | 0.019 |
| BMI | rs3888190 | *SH2B1* | Yes | Associated with lots of other traits and is a big haplotype | A | C | 0.999946 | 0.031 |
| BMI | rs4256980 | *TRIM66* | No | NA | G | C | 0.996389 | 0.021 |
| BMI | rs4740619 | *C9orf93* | No | NA | T | C | 0.999082 | 0.018 |
| BMI | rs543874 | *SEC16B* | No | NA | G | A | 1 | 0.048 |
| BMI | rs6477694 | *EPB41L4B* | No | NA | C | T | 0.992092 | 0.017 |
| BMI | rs6567160 | *MC4R* | No | NA | C | T | 0.998876 | 0.056 |
| BMI | rs657452 | *AGBL4* | No | NA | A | G | 0.988962 | 0.023 |
| BMI | rs6804842 | *RARB* | No | NA | G | A | 0.993341 | 0.019 |
| BMI | rs7138803 | *BCDIN3D* | No | NA | A | G | 1 | 0.032 |
| BMI | rs7141420 | *NRXN3* | No | NA | T | C | 0.988677 | 0.024 |
| BMI | rs7243357 | *GRP* | No | NA | T | G | 0.9922 | 0.022 |
| BMI | rs758747 | *NLRC3* | No | NA | T | C | 0.979448 | 0.023 |
| BMI | rs7599312 | *ERBB4* | No | NA | G | A | 0.981254 | 0.022 |
| BMI | rs7899106 | *GRID1* | No | NA | G | A | 0.990754 | 0.04 |
| BMI | rs9400239 | *FOXO3* | No | NA | C | T | 0.995847 | 0.019 |
| BMI | rs9581854 | *MTIF3* | No | NA | T | C | 0.996839 | 0.03 |
| BMI | rs9925964 | *KAT8* | No | NA | A | G | 0.998274 | 0.019 |

**Supplementary Table S2:** Summary of the results from the multiple methods of Mendelian randomisation in Caucasian individuals.

1. Linear and logistic regression, MR using 73 SNP BMI GRS

1. Results from 942 SNP GRS, and results from 73 SNP IVW analysis
2. MR Egger and Median IV results

**Supplementary Table S3. Adjustment of observational results for maternal smoking and birth weight**

|  |  |  | Age and sex adjusted |  |  |  | Adjust for early life factors - maternal smoking and birth weight |  |  |
| --- | --- | --- | --- | --- | --- | --- | --- | --- | --- |
|  |  |  | RESULTS FROM LINEAR AND LOGISTIC REGRESSION ANALYSES |  |  |  | RESULTS FROM LINEAR AND LOGISTIC REGRESSION ANALYSES |  |  |
| Outcome | Strata | Number | Difference in outcome (95% CI) per SD higher BMI* | P value | P compare men and women | Number | Difference in outcome (95% CI) per SD higher BMI* | P value | P compare men and women |
|  |  |  | Socioeconomic position measures |  |  |  | Socioeconomic position measures |  |  |
| Townsend deprivation index | All | 377786 | 0.079 (0.076, 0.082) | <1x10-15 |  | 162932 | 0.074 (0.069, 0.079) | <1x10-15 |  |
| Townsend deprivation index | Men only | 174144 | 0.049 (0.045, 0.054) | <1x10-15 | <1x10-15 | 63932 | 0.043 (0.035, 0.051) | <1x10-15 | <1x10-15 |
| Townsend deprivation index | Women only | 203642 | 0.104 (0.100, 0.108) | <1x10-15 |  | 99000 | 0.095 (0.089, 0.101) | <1x10-15 |  |
| Annual household income | All | 326117 | -0.073 (-0.077, -0.070) | <1x10-15 |  | 144484 | -0.065 (-0.071, -0.060) | <1x10-15 |  |
| Annual household income | Men only | 156521 | -0.030 (-0.040, -0.020) | <1x10-15 | <1x10-15 | 58931 | -0.011 (-0.020, -0.003) | 0.007 | <1x10-15 |
| Annual household income | Women only | 169596 | -0.113 (-0.118, -0.108) | <1x10-15 |  | 85553 | -0.102 (-0.109, -0.096) | <1x10-15 |  |
| Years in education | All | 374658 | -0.100 (-0.103, -0.098) | <1x10-15 |  | 162042 | -0.086 (-0.091, -0.081) | <1x10-15 |  |
| Years in education | Men only | 172637 | -0.108 (-0.112, -0.103) | <1x10-15 | 0.00003 | 63566 | -0.092 (-0.100, -0.086) | <1x10-15 | 0.010 |
| Years in education | Women only | 202021 | -0.095 (-0.099, -0.090) | <1x10-15 |  | 98476 | -0.081 (-0.086, -0.075) | <1x10-15 |  |
| Degree level education | All | 374658 | OR: 0.82 (0.81, 0.82) | <1x10-15 |  | 162042 | OR: 0.82 (0.82, 0.83) | <1x10-15 |  |
| Degree level education | Men only | 172637 | OR: 0.82 (0.81, 0.82) | <1x10-15 | 0.60 | 63566 | OR: 0.82 (0.80, 0.83) | <1x10-15 | 0.18 |
| Degree level education | Women only | 202021 | OR: 0.82 (0.81, 0.83) | <1x10-15 |  | 98476 | OR: 0.83 (0.82, 0.84) | <1x10-15 |  |
| Have a skilled job | All | 245138 | OR: 0.87 (0.86, 0.88) | <1x10-15 |  | 113945 | OR: 0.88 (0.86, 0.89) | <1x10-15 |  |
| Have a skilled job | Men only | 117866 | OR: 0.87 (0.86, 0.89) | <1x10-15 | 0.30 | 47287 | OR: 0.87 (0.84, 0.89) | <1x10-15 | 0.23 |
| Have a skilled job | Women only | 127272 | OR: 0.86 (0.85, 0.87) | <1x10-15 |  | 66658 | OR: 0.88 (0.86, 0.90) | <1x10-15 |  |
| In employment | All | 222288 | OR: 0.92 (0.90, 0.85) | 1x10-9 |  | 105851 | OR: 0.93 (0.89, 0.97) | 0.0007 |  |
| In employment | Men only | 109002 | OR: 0.99 (0.96, 1.02) | 0.54 | <1x10-15 | 44862 | OR: 1.01 (0.96, 1.07) | 0.59 | 2x10-6 |
| In employment | Women only | 113286 | OR: 0.81 (0.77, 0.84) | <1x10-15 |  | 113286 | OR: 0.83 (0.78, 0.88) | <1x10-15 |  |
|  |  |  | Social support measures |  |  |  | Social support measures |  |  |
| Weekly visits from friends and family | All | 375985 | OR: 1.06 (1.05, 1.07) | <1x10-15 |  | 162452 | OR: 1.06 (1.05, 1.08) | <1x10-15 |  |
| Weekly visits from friends and family | Men only | 173165 | OR: 1.08 (1.07, 1.09) | <1x10-15 | 7x10-5 | 63711 | OR: 1.08 (1.06, 1.10) | 2x10-15 | 0.11 |
| Weekly visits from friends and family | Women only | 202820 | OR: 1.04 (1.03, 1.06) | 3x10-13 |  | 98741 | OR: 1.05 (1.03, 1.07) | 7x10-9 |  |
| Weekly participation in leisure and social activities | All | 377246 | OR: 0.91 (0.90, 0.91) | <1x10-15 |  | 162841 | OR: 0.90 (0.89, 0.91) | <1x10-15 |  |
| Weekly participation in leisure and social activities | Men only | 173923 | OR: 0.97 (0.96, 0.98) | 1x10-10 | <1x10-15 | 63934 | OR: 0.96 (0.95, 0.98) | 4x10-5 | <1x10-15 |
| Weekly participation in leisure and social activities | Women only | 203323 | OR: 0.86 (0.85, 0.87) | <1x10-15 |  | 98907 | OR: 0.86 (0.85, 0.87) | <1x10-15 |  |
| Weekly confiding in close friend or relative | All | 367216 | OR: 0.96 (0.95, 0.96) | <1x10-15 |  | 159619 | OR: 0.96 (0.95, 0.97) | <1x10-15 |  |
| Weekly confiding in close friend or relative | Men only | 168707 | OR: 0.97 (0.96, 0.98) | 2x10-7 | 3x10-5 | 62521 | OR: 0.98 (0.96, 1.00) | 0.019 | 0.0003 |
| Weekly confiding in close friend or relative | Women only | 198509 | OR: 0.94 (0.93, 0.95) | <1x10-15 |  | 97098 | OR: 0.94 (0.93, 0.96) | 2x10-13 |  |
| Cohabitation with partner or spouse | All | 347329 | OR: 0.98 (0.97, 0.99) | 3x10-5 |  | 148674 | OR: 0.97 (0.96, 0.99) | 0.0001 |  |
| Cohabitation with partner or spouse | Men only | 163873 | OR: 1.04 (1.03, 1.05) | 6x10-10 | <1x10-15 | 59926 | OR: 1.04 (1.03, 1.07) | 9x10-6 | <1x10-15 |
| Cohabitation with partner or spouse | Women only | 183456 | OR: 0.94 (0.93, 0.96) | <1x10-15 |  | 88748 | OR: 0.93 (0.92, 0.95) | <1x10-15 |  |

| **Supplementary Table S4:** Associations between BMI and death of a partner/spouse or marital separation/divorce | | | |  |
| --- | --- | --- | --- | --- |
|  |  |  |  |  |
| **Outcome** | **Individuals included** | **N Cases - controls** | **OR (95%CI) of outcome per SD higher BMI** | **P value** |
| Divorce / marital separation in last 2 years | All | 7,745 (339,536) | 0.98 (0.96, 1.00) | 0.03 |
| Divorce / marital separation in last 2 years | Men only | 4,047 (159,808) | 1.02 (0.99, 1.05) | 0.14 |
| Divorce / marital separation in last 2 years | Women only | 3,698 (179,728) | 0.95 (0.92, 0.97) | 2x10-5 |
| Death of partner or spouse in last 2 years | All | 4,415 (342,866) | 1.05 (1.02, 1.07) | 0.001 |
| Death of partner or spouse in last 2 years | Men only | 1,463 (162,392) | 1.07 (1.02, 1.12) | 0.004 |
| Death of partner or spouse in last 2 years | Women only | 2,952 (180,474) | 1.03 (1.00, 1.07) | 0.036 |

| **Supplementary Table S5:** Association between BMI and cohabiting with a partner or spouse, stratified by age | | | | |  |  |  |
| --- | --- | --- | --- | --- | --- | --- | --- |
|  |  |  |  |  |  |  |  |
|  |  |  |  | **Logistic regression** |  | **Mendelian Randomization** |  |
| **Outcome** | **Individuals included** | **Age** | **N Cases - controls** | **OR (95%CI) of outcome per SD higher BMI** | **P value** | **OR (95%CI) of outcome per SD higher BMI** | **P value** |
| Cohabitation with a partner or spouse | All | <58 years | 130,804 (28,934) | 0.98 (0.97, 1.00) | 0.014 | 0.89 (0.81, 0.98) | 0.014 |
| Cohabitation with a partner or spouse | Men only | <58 years | 60,140 (14,004) | 1.08 (1.06, 1.10) | 4x10-15 | 1.07 (0.94, 1.21) | 0.3 |
| Cohabitation with a partner or spouse | Women only | <58 years | 70,664 (14,930) | 0.91 (0.89, 0.92) | 2x10-27 | 0.74 (0.64, 0.85) | 1x10-5 |
| Cohabitation with a partner or spouse | All | >58 years | 146,595 (40,996) | 0.98 (0.97, 0.99) | 0.0007 | 0.99 (0.90, 1.09) | 0.88 |
| Cohabitation with a partner or spouse | Men only | >58 years | 73,922 (15,807) | 1.01 (0.99, 1.03) | 0.31 | 1.08 (0.94, 1.24) | 0.27 |
| Cohabitation with a partner or spouse | Women only | >58 years | 72,673 (25,189) | 0.96 (0.95, 0.98) | 1x10-7 | 0.93 (0.81, 1.06) | 0.27 |

| **Supplementary Table S6:** Genetic associations between BMI and annual household income with and without adjustment for number of people living in the household | | | | | |  |
| --- | --- | --- | --- | --- | --- | --- |
|  |  |  | **RESULTS FROM LINEAR REGRESSION MODELS** | | **RESULTS FROM 1-SAMPLE 73 SNP BMI GRS** | |
| **Individuals** | **Adjustment** | **N** | **Change in outcome (95% CI) per SD higher BMI*** | **P value** | **Change in outcome (95% CI) per SD higher BMI**** | **P value** |
| All | Standard | 326,117 | -0.07 (-0.07, -0.06) | <1x10-15 | -0.08 (-0.10, -0.06) | 4x10-7 |
| All | Standard + number living in household | 325,782 | -0.06 (-0.07, -0.06) | <1x10-15 | -0.06 (-0.09, -0.04) | 4x10-6 |
| Men only | Standard | 156,521 | -0.03 (-0.03, -0.02) | <1x10-15 | -0.05 (-0.09, -0.01) | 0.006 |
| Men only | Standard + number living in household | 156,385 | -0.03 (-0.03, -0.02) | <1x10-15 | -0.05 (-0.09, -0.01) | 0.006 |
| Women only | Standard | 169,596 | -0.10 (-0.11, -0.10) | <1x10-15 | -0.09 (-0.12, -0.05) | 9x10-6 |
| Women only | Standard + number living in household | 169,397 | -0.10 (-0.10, -0.09) | <1x10-15 | -0.07 (-0.11, -0.03) | 0.0002 |

| **Supplementary Table S7:** Observational and genetic associations between BMI and the different outcome measures in a subset of individuals with no known health problems. | | | | | |  |
| --- | --- | --- | --- | --- | --- | --- |
|  |  |  | **RESULTS FROM LINEAR AND LOGISTIC REGRESSION MODELS** | | **RESULTS FROM 1-SAMPLE 73 SNP BMI GRS** | |
| **Outcome** | **Strata** | **Number** | **Change in outcome (95% CI) per SD higher BMI*** | **P value** | **Change in outcome (95% CI) per SD higher BMI**** | **P value** |
| **Socioeconomic position measures** | | | | | | |
| Townsend deprivation index | All | 88,223 | 0.04 (0.03, 0.05) | <1x10-15 | 0.06 (0.01, 0.12) | 0.031 |
| Townsend deprivation index | Men only | 41,222 | 0.007 (-0.003, 0.018) | 0.17 | 0.03 (-0.05, 0.11) | 0.44 |
| Townsend deprivation index | Women only | 47,001 | 0.07 (0.06, 0.08) | <1x10-15 | 0.09 (0.01, 0.17) | 0.024 |
| Annual household income | All | 78,158 | -0.03 (-0.04, -0.02) | 2x10-14 | -0.03 (-0.09, 0.03) | 0.29 |
| Annual household income | Men only | 37,672 | 0.03 (0.02, 0.04) | 1x10-6 | -0.01 (-0.09, 0.07) | 0.82 |
| Annual household income | Women only | 40,486 | -0.08 (-0.09, -0.07) | <1x10-15 | -0.05 (-0.14, 0.03) | 0.22 |
| Years in education | All | 87,549 | -0.09 (-0.10, -0.09) | <1x10-15 | -0.02 (-0.08, 0.03) | 0.42 |
| Years in education | Men only | 40,889 | -0.10 (-0.11, -0.09) | <1x10-15 | -0.03 (-0.11, 0.04) | 0.38 |
| Years in education | Women only | 46,660 | -0.09 (-0.09, -0.08) | <1x10-15 | -0.01 (-0.09, 0.06) | 0.74 |
| Degree level education | All | 87,549 | OR: 0.82 (0.80, 0.83) | <1x10-15 | OR: 0.93 (0.83, 1.05) | 0.25 |
| Degree level education | Men only | 40,889 | OR: 0.82 (0.80, 0.83) | <1x10-15 | OR: 0.94 (0.79, 1.11) | 0.45 |
| Degree level education | Women only | 46,660 | OR: 0.82 (0.80, 0.84) | <1x10-15 | OR: 0.93 (0.79, 1.10) | 0.41 |
| Have a skilled job | All | 67,684 | OR: 0.87 (0.85, 0.89) | <1x10-15 | OR: 0.95 (0.80, 1.13) | 0.54 |
| Have a skilled job | Men only | 33,104 | OR: 0.87 (0.85, 0.90) | <1x10-15 | OR: 1.04 (0.81, 1.33) | 0.76 |
| Have a skilled job | Women only | 34,580 | OR: 0.86 (0.84, 0.89) | <1x10-15 | OR: 0.88 (0.69, 1.12) | 0.30 |
| In employment | All | 65,298 | OR: 1.00 (0.94, 1.06) | 0.97 | OR: 0.77 (0.49, 1.21) | 0.26 |
| In employment | Men only | 32,387 | OR: 1.12 (1.05, 1.21) | 0.0013 | OR: 0.74 (0.43, 1.27) | 0.28 |
| In employment | Women only | 32,911 | OR: 0.77 (0.70, 0.86) | 1x10-6 | OR: 0.87 (0.40, 1.87) | 0.71 |
| **Social support measures** | | | | | | |
| Weekly visits from friends and family | All | 87,750 | OR: 1.09 (1.07, 1.11) | <1x10-15 | OR: 0.91 (0.79, 1.05) | 0.19 |
| Weekly visits from friends and family | Men only | 40,971 | OR: 1.13 (1.11, 1.16) | <1x10-15 | OR: 0.87 (0.72, 1.05) | 0.14 |
| Weekly visits from friends and family | Women only | 46,779 | OR: 1.04 (1.01, 1.07) | 0.003 | OR: 0.96 (0.77, 1.18) | 0.68 |
| Weekly participation in leisure and social activities | All | 88,115 | OR: 0.94 (0.92, 0.95) | 3x10-15 | OR: 1.00 (0.88, 1.14) | 0.99 |
| Weekly participation in leisure and social activities | Men only | 41,170 | OR: 1.01 (0.99, 1.03) | 0.39 | OR: 0.99 (0.83, 1.18) | 0.90 |
| Weekly participation in leisure and social activities | Women only | 46,945 | OR: 0.87 (0.86, 0.89) | <1x10-15 | OR: 1.00 (0.84, 1.21) | 0.96 |
| Weekly confiding in close friend or relative | All | 85,887 | OR: 0.99 (0.97, 1.00) | 0.15 | OR: 1.02 (0.89, 1.17) | 0.77 |
| Weekly confiding in close friend or relative | Men only | 40,036 | OR: 1.00 (0.98, 1.02) | 0.92 | OR: 0.96 (0.80, 1.15) | 0.65 |
| Weekly confiding in close friend or relative | Women only | 45,851 | OR: 0.98 (0.95, 1.00) | 0.08 | OR: 1.11 (0.90, 1.37) | 0.34 |
| Cohabitation with partner or spouse | All | 80,782 | OR: 1.03 (1.01, 1.05) | 0.004 | OR: 1.00 (0.85, 1.18) | 0.99 |
| Cohabitation with partner or spouse | Men only | 38,698 | OR: 1.13 (1.10, 1.16) | 1x10-15 | OR: 1.06 (0.84, 1.33) | 0.65 |
| Cohabitation with partner or spouse | Women only | 42,084 | OR: 0.96 (0.93, 0.99) | 0.004 | OR: 0.96 (0.90, 1.37) | 0.74 |

| **Supplementary Table S8**: results from non-linear MR across deciles of IV-free BMI | | | | | |  |
| --- | --- | --- | --- | --- | --- | --- |
|  |  |  |  |  |  |  |
| **Outcome** | **Individuals** | **Decile** | **Beta** | **Standard error** | **P value** | **BMI range within the decile** |
| TDI | All | 1 | -0.047 | 0.009 | 6x10-8 | 12.0 to 22.0 kg/m2 |
| TDI | All | 2 | 0.003 | 0.008 | 0.75 | 22.0 to 23.5 kg/m2 |
| TDI | All | 3 | -0.001 | 0.008 | 0.87 | 23.5 to 24.6 kg/m2 |
| TDI | All | 4 | 0.014 | 0.008 | 0.09 | 26.4 to 25.7 kg/m2 |
| TDI | All | 5 | 0.036 | 0.008 | 2x10-5 | 25.7 to 26.7 kg/m2 |
| TDI | All | 6 | 0.016 | 0.008 | 0.05 | 26.7 to 27.8 kg/m2 |
| TDI | All | 7 | 0.046 | 0.008 | 5x10-8 | 27.8 to 29.1 kg/m2 |
| TDI | All | 8 | 0.017 | 0.009 | 0.043 | 29.1 to 30.7 kg/m2 |
| TDI | All | 9 | 0.009 | 0.009 | 0.28 | 30.7 to 33.4 kg/m2 |
| TDI | All | 10 | 0.040 | 0.009 | 5x10-6 | 33.4 to 74.7 kg/m2 |
| TDI | Men only | 1 | -0.064 | 0.014 | 4x10-6 | 12.0 to 22.0 kg/m2 |
| TDI | Men only | 2 | -0.008 | 0.013 | 0.567 | 22.0 to 23.5 kg/m2 |
| TDI | Men only | 3 | -0.009 | 0.013 | 0.49 | 23.5 to 24.6 kg/m2 |
| TDI | Men only | 4 | 0.012 | 0.013 | 0.35 | 26.4 to 25.7 kg/m2 |
| TDI | Men only | 5 | 0.025 | 0.013 | 0.06 | 25.7 to 26.7 kg/m2 |
| TDI | Men only | 6 | 0.009 | 0.013 | 0.51 | 26.7 to 27.8 kg/m2 |
| TDI | Men only | 7 | 0.061 | 0.013 | 4x10-6 | 27.8 to 29.1 kg/m2 |
| TDI | Men only | 8 | 0.015 | 0.013 | 0.26 | 29.1 to 30.7 kg/m2 |
| TDI | Men only | 9 | 0.020 | 0.013 | 0.14 | 30.7 to 33.4 kg/m2 |
| TDI | Men only | 10 | 0.040 | 0.014 | 0.003 | 33.4 to 74.7 kg/m2 |
| TDI | Women only | 1 | -0.031 | 0.011 | 0.007 | 12.0 to 22.0 kg/m2 |
| TDI | Women only | 2 | 0.002 | 0.011 | 0.85 | 22.0 to 23.5 kg/m2 |
| TDI | Women only | 3 | 0.012 | 0.011 | 0.27 | 23.5 to 24.6 kg/m2 |
| TDI | Women only | 4 | 0.013 | 0.011 | 0.22 | 26.4 to 25.7 kg/m2 |
| TDI | Women only | 5 | 0.043 | 0.011 | 1x10-4 | 25.7 to 26.7 kg/m2 |
| TDI | Women only | 6 | 0.028 | 0.011 | 0.010 | 26.7 to 27.8 kg/m2 |
| TDI | Women only | 7 | 0.048 | 0.011 | 1x10-5 | 27.8 to 29.1 kg/m2 |
| TDI | Women only | 8 | 0.006 | 0.011 | 0.60 | 29.1 to 30.7 kg/m2 |
| TDI | Women only | 9 | 0.013 | 0.011 | 0.27 | 30.7 to 33.4 kg/m2 |
| TDI | Women only | 10 | 0.030 | 0.011 | 0.009 | 33.4 to 74.7 kg/m2 |
| Income | All | 1 | 0.066 | 0.010 | 2x10-10 | 12.0 to 22.0 kg/m2 |
| Income | All | 2 | 0.023 | 0.010 | 0.022 | 22.0 to 23.5 kg/m2 |
| Income | All | 3 | -0.013 | 0.010 | 0.20 | 23.5 to 24.6 kg/m2 |
| Income | All | 4 | -0.023 | 0.010 | 0.019 | 26.4 to 25.7 kg/m2 |
| Income | All | 5 | -0.034 | 0.010 | 0.0006 | 25.7 to 26.7 kg/m2 |
| Income | All | 6 | -0.028 | 0.010 | 0.005 | 26.7 to 27.8 kg/m2 |
| Income | All | 7 | -0.033 | 0.010 | 0.0008 | 27.8 to 29.1 kg/m2 |
| Income | All | 8 | -0.019 | 0.010 | 0.05 | 29.1 to 30.7 kg/m2 |
| Income | All | 9 | -0.020 | 0.010 | 0.040 | 30.7 to 33.4 kg/m2 |
| Income | All | 10 | -0.031 | 0.010 | 0.001 | 33.4 to 74.7 kg/m2 |
| Income | Men only | 1 | 0.094 | 0.016 | 4x10-9 | 12.0 to 22.0 kg/m2 |
| Income | Men only | 2 | 0.024 | 0.016 | 0.13 | 22.0 to 23.5 kg/m2 |
| Income | Men only | 3 | -0.012 | 0.016 | 0.45 | 23.5 to 24.6 kg/m2 |
| Income | Men only | 4 | -0.007 | 0.015 | 0.63 | 26.4 to 25.7 kg/m2 |
| Income | Men only | 5 | -0.016 | 0.016 | 0.30 | 25.7 to 26.7 kg/m2 |
| Income | Men only | 6 | -0.036 | 0.016 | 0.021 | 26.7 to 27.8 kg/m2 |
| Income | Men only | 7 | -0.021 | 0.016 | 0.17 | 27.8 to 29.1 kg/m2 |
| Income | Men only | 8 | -0.031 | 0.015 | 0.040 | 29.1 to 30.7 kg/m2 |
| Income | Men only | 9 | -0.021 | 0.016 | 0.18 | 30.7 to 33.4 kg/m2 |
| Income | Men only | 10 | -0.042 | 0.016 | 0.007 | 33.4 to 74.7 kg/m2 |
| Income | Women only | 1 | 0.051 | 0.014 | 0.0002 | 12.0 to 22.0 kg/m2 |
| Income | Women only | 2 | 0.004 | 0.013 | 0.76 | 22.0 to 23.5 kg/m2 |
| Income | Women only | 3 | -0.009 | 0.013 | 0.49 | 23.5 to 24.6 kg/m2 |
| Income | Women only | 4 | -0.030 | 0.013 | 0.019 | 26.4 to 25.7 kg/m2 |
| Income | Women only | 5 | -0.042 | 0.013 | 0.001 | 25.7 to 26.7 kg/m2 |
| Income | Women only | 6 | -0.041 | 0.013 | 0.001 | 26.7 to 27.8 kg/m2 |
| Income | Women only | 7 | -0.036 | 0.013 | 0.005 | 27.8 to 29.1 kg/m2 |
| Income | Women only | 8 | -0.008 | 0.013 | 0.54 | 29.1 to 30.7 kg/m2 |
| Income | Women only | 9 | -0.009 | 0.013 | 0.47 | 30.7 to 33.4 kg/m2 |
| Income | Women only | 10 | -0.033 | 0.012 | 0.007 | 33.4 to 74.7 kg/m2 |
| Cohabitation | All | 1 | 0.073 | 0.018 | 7x10-5 | 12.0 to 22.0 kg/m2 |
| Cohabitation | All | 2 | 0.076 | 0.019 | 7x10-5 | 22.0 to 23.5 kg/m2 |
| Cohabitation | All | 3 | 0.036 | 0.019 | 0.06 | 23.5 to 24.6 kg/m2 |
| Cohabitation | All | 4 | 0.008 | 0.020 | 0.69 | 26.4 to 25.7 kg/m2 |
| Cohabitation | All | 5 | -0.015 | 0.020 | 0.45 | 25.7 to 26.7 kg/m2 |
| Cohabitation | All | 6 | -0.013 | 0.020 | 0.52 | 26.7 to 27.8 kg/m2 |
| Cohabitation | All | 7 | -0.016 | 0.020 | 0.40 | 27.8 to 29.1 kg/m2 |
| Cohabitation | All | 8 | -0.027 | 0.019 | 0.16 | 29.1 to 30.7 kg/m2 |
| Cohabitation | All | 9 | -0.034 | 0.019 | 0.07 | 30.7 to 33.4 kg/m2 |
| Cohabitation | All | 10 | -0.071 | 0.017 | 5x10-5 | 33.4 to 74.7 kg/m2 |
| Cohabitation | Men only | 1 | 0.183 | 0.027 | 1x10-11 | 12.0 to 22.0 kg/m2 |
| Cohabitation | Men only | 2 | 0.068 | 0.029 | 0.020 | 22.0 to 23.5 kg/m2 |
| Cohabitation | Men only | 3 | 0.096 | 0.030 | 0.002 | 23.5 to 24.6 kg/m2 |
| Cohabitation | Men only | 4 | 0.050 | 0.031 | 0.10 | 26.4 to 25.7 kg/m2 |
| Cohabitation | Men only | 5 | -0.012 | 0.031 | 0.71 | 25.7 to 26.7 kg/m2 |
| Cohabitation | Men only | 6 | -0.015 | 0.031 | 0.63 | 26.7 to 27.8 kg/m2 |
| Cohabitation | Men only | 7 | 0.004 | 0.031 | 0.89 | 27.8 to 29.1 kg/m2 |
| Cohabitation | Men only | 8 | -0.044 | 0.030 | 0.15 | 29.1 to 30.7 kg/m2 |
| Cohabitation | Men only | 9 | 0.006 | 0.030 | 0.83 | 30.7 to 33.4 kg/m2 |
| Cohabitation | Men only | 10 | -0.047 | 0.027 | 0.08 | 33.4 to 74.7 kg/m2 |
| Cohabitation | Women only | 1 | 0.022 | 0.025 | 0.38 | 12.0 to 22.0 kg/m2 |
| Cohabitation | Women only | 2 | 0.046 | 0.026 | 0.08 | 22.0 to 23.5 kg/m2 |
| Cohabitation | Women only | 3 | 0.035 | 0.026 | 0.17 | 23.5 to 24.6 kg/m2 |
| Cohabitation | Women only | 4 | -0.031 | 0.026 | 0.22 | 26.4 to 25.7 kg/m2 |
| Cohabitation | Women only | 5 | -0.011 | 0.025 | 0.65 | 25.7 to 26.7 kg/m2 |
| Cohabitation | Women only | 6 | -0.023 | 0.025 | 0.35 | 26.7 to 27.8 kg/m2 |
| Cohabitation | Women only | 7 | -0.028 | 0.025 | 0.26 | 27.8 to 29.1 kg/m2 |
| Cohabitation | Women only | 8 | -0.045 | 0.025 | 0.07 | 29.1 to 30.7 kg/m2 |
| Cohabitation | Women only | 9 | -0.050 | 0.024 | 0.037 | 30.7 to 33.4 kg/m2 |
| Cohabitation | Women only | 10 | -0.085 | 0.023 | 0.0002 | 33.4 to 74.7 kg/m2 |

| **Supplementary Table S9:** Summary of the results from the non-linear Mendelian randomisation analyses for all outcome traits against BMI | | | | | |  |  |
| --- | --- | --- | --- | --- | --- | --- | --- |
|  |  |  |  | **Evidence of non-linearity from Fractional Polynomial model** | | **Evidence of non-linearity from Piecewise Linear model** | |
| **Outcome** | **Strata** | **Number** | **IV P-value from the Fractional Polynomial model** | **Fractional polynomial test** | **Cochran Q test** | **Quadratic test** | **Cochran Q** |
| **Socioeconomic position measures** | | | | | | | |
| Townsend deprivation index | All | 377,786 | <1x10-15 | 0.00028 | 7x10-8 | 3x10-9 | 2x10-14 |
| Townsend deprivation index | Men only | 174,144 | 5x10-12 | 0.016 | 1x10-5 | 2x10-7 | 2x10-8 |
| Townsend deprivation index | Women only | 203,642 | 7x10-8 | 0.021 | 0.01 | 0.006 | 3x10-5 |
| Annual household income | All | 326,117 | <1x10-15 | 0.0006 | 8x10-13 | 4x10-9 | 5x10-15 |
| Annual household income | Men only | 156,521 | 1x10-15 | 0.04 | 3x10-8 | 2x10-9 | 5x10-9 |
| Annual household income | Women only | 169,596 | 8x10-10 | 0.02 | 0.003 | 0.004 | 5x10-6 |
| Years in education | All | 374,658 | 0.0001 | 0.48 | 0.34 | 0.13 | 0.22 |
| Years in education | Men only | 172,637 | 0.0004 | 0.99 | 0.54 | 0.96 | 0.44 |
| Years in education | Women only | 202,021 | 0.02 | 0.14 | 0.88 | 0.031 | 0.25 |
| Degree level education | All | 374,658 | 2x10-5 | 0.13 | 0.68 | 0.07 | 0.53 |
| Degree level education | Men only | 172,637 | 0.001 | 0.73 | 0.06 | 0.82 | 0.04 |
| Degree level education | Women only | 202,021 | 0.005 | 0.09 | 0.14 | 0.05 | 0.34 |
| Have a skilled job | All | 245,138 | 0.0031 | 0.53 | 0.79 | 0.49 | 0.26 |
| Have a skilled job | Men only | 117,866 | 0.005 | 0.14 | 0.5 | 0.16 | 0.3 |
| Have a skilled job | Women only | 127,272 | 0.04 | 0.87 | 0.28 | 0.72 | 0.41 |
| In employment* | All | 222,288 |  |  |  |  |  |
| In employment* | Men only | 109,002 |  |  |  |  |  |
| In employment* | Women only | 113,286 |  |  |  |  |  |
| **Social support measures** | | | | | | | |
| Weekly visits from friends and family | All | 375,985 | 0.034 | 0.06 | 0.034 | 0.035 | 0.1 |
| Weekly visits from friends and family | Men only | 173,165 | 0.33 | 0.33 | 0.042 | 0.058 | 0.42 |
| Weekly visits from friends and family | Women only | 202,820 | 0.077 | 0.13 | 0.5 | 0.31 | 0.43 |
| Weekly participation in leisure and social activities | All | 377,246 | 0.001 | 0.001 | 1x10-7 | 4x10-10 | 2x10-11 |
| Weekly participation in leisure and social activities | Men only | 173,923 | 0.014 | 0.044 | 0.14 | 4x10-6 | 1x10-5 |
| Weekly participation in leisure and social activities | Women only | 203,323 | 0.003 | 0.003 | 0.009 | 2x10-5 | 1x10-6 |
| Weekly confiding in close friend or relative | All | 367,216 | 0.098 | 0.12 | 0.08 | 0.19 | 0.14 |
| Weekly confiding in close friend or relative | Men only | 168,707 | 0.097 | 0.12 | 0.32 | 0.13 | 0.035 |
| Weekly confiding in close friend or relative | Women only | 198,509 | 0.46 | 0.52 | 0.65 | 0.63 | 0.94 |
| Cohabitation with partner or spouse | All | 347,329 | 1x10-5 | 2x10-5 | 0.0005 | 1x10-12 | 2x10-9 |
| Cohabitation with partner or spouse | Men only | 163,873 | 1x10-11 | 4x10-8 | 0.0009 | 4x10-13 | 4x10-9 |
| Cohabitation with partner or spouse | Women only | 183,456 | 0.0003 | 0.003 | 0.024 | 7x10-6 | 0.003 |
| *Greyed out as no results available because the outcome is very unbalanced which meant the models were unable to run | | | | |  |  |  |

**Supplementary Table S10. Results of within-family analyses compared to MR in unrelated individuals**

| **Outcome** | **Strata** | **Type of analysis** | **Number** | **Difference in outcome (95% CI) per SD higher BMI*** | **P value** |
| --- | --- | --- | --- | --- | --- |
|  |  |  |  |  |  |
| Townsend deprivation index | All | MR in unrelated individuals | 379209 | 0.079 (0.055, 0.103) | 7x10-11 |
| Townsend deprivation index | All | Siblings [Non-genetic, family effects] | 39865 | 0.047 (0.031, 0.062) | 3x10-9 |
| Townsend deprivation index | All | Siblings [MR, family effects] | 39865 | 0.021 (-0.136, 0.178) | 0.79 |
| Townsend deprivation index | Men only | MR in unrelated individuals | 174059 | 0.077 (0.039, 0.116) | 0.00008 |
| Townsend deprivation index | Men only | Siblings [Non-genetic, family effects] | 16777 | -0.006 (-0.045, 0.034) | 0.78 |
| Townsend deprivation index | Men only | Siblings [MR, family effects] | 16777 | -0.135 (-0.525, 0.254) | 0.50 |
| Townsend deprivation index | Women only | MR in unrelated individuals | 205150 | 0.081 (0.051, 0.111) | 1x10-7 |
| Townsend deprivation index | Women only | Siblings [Non-genetic, family effects] | 23088 | 0.078 (0.054, 0.101) | 5x10-7 |
| Townsend deprivation index | Women only | Siblings [MR, family effects] | 23088 | 0.234 (-0.006, 0.474) | 0.06 |
| Annual household income | All | MR in unrelated individuals | 327244 | -0.067 (-0.093, -0.042) | 1x10-7 |
| Annual household income | All | Siblings [Non-genetic, family effects] | 34523 | -0.027 (-0.043, -0.011) | 0.001 |
| Annual household income | All | Siblings [MR, family effects] | 34523 | 0.112 (-0.059, 0.283) | 0.20 |
| Annual household income | Men only | MR in unrelated individuals | 156467 | -0.044 (-0.084, -0.004) | 0.029 |
| Annual household income | Men only | Siblings [Non-genetic, family effects] | 15172 | 0.015 (-0.024, 0.055) | 0.45 |
| Annual household income | Men only | Siblings [MR, family effects] | 15172 | 0.364 (-0.076, 0.804) | 0.11 |
| Annual household income | Women only | MR in unrelated individuals | 170777 | -0.085 (-0.118, -0.053) | 2x10-7 |
| Annual household income | Women only | Siblings [Non-genetic, family effects] | 19351 | -0.042 (-0.068, -0.017) | 0.001 |
| Annual household income | Women only | Siblings [MR, family effects] | 19351 | -0.039 (-0.282, 0.204) | 0.75 |
| Years in education | All | MR in unrelated individuals | 375950 | -0.047 (-0.071, -0.022) | 0.0002 |
| Years in education | All | Siblings [Non-genetic, family effects] | 39570 | -0.028 (-0.044, -0.012) | 0.0007 |
| Years in education | All | Siblings [MR, family effects] | 39570 | -0.009 (-0.171, 0.154) | 0.91 |
| Years in education | Men only | MR in unrelated individuals | 172486 | -0.055 (-0.094, -0.015) | 0.020 |
| Years in education | Men only | Siblings [Non-genetic, family effects] | 16640 | -0.070 (-0.111, -0.029) | 0.0007 |
| Years in education | Men only | Siblings [MR, family effects] | 16640 | 0.010 (-0.377, 0.398) | 0.96 |
| Years in education | Women only | MR in unrelated individuals | 203464 | -0.041 (-0.072, 0.010) | 0.010 |
| Years in education | Women only | Siblings [Non-genetic, family effects] | 22930 | -0.015 (-0.040, 0.009) | 0.22 |
| Years in education | Women only | Siblings [MR, family effects] | 22930 | -0.020 (-0.265, 0.225) | 0.87 |
| Degree | All | MR in unrelated individuals | 375950 | -0.082 (-0.134, -0.030) | 0.0002 |
| Degree | All | Siblings [Non-genetic, family effects] | 39335 | -0.047 (-0.081, -0.014) | 0.006 |
| Degree | All | Siblings [MR, family effects] | 39335 | -0.029 (-0.381, 0.323) | 0.87 |
| Degree | Men only | MR in unrelated individuals | 172486 | -0.085 (-0.168, -0.003) | 0.043 |
| Degree | Men only | Siblings [Non-genetic, family effects] | 8007 | -0.072 (-0.156, 0.013) | 0.10 |
| Degree | Men only | Siblings [MR, family effects] | 8007 | -0.166 (-0.968, 0.637) | 0.69 |
| Degree | Women only | MR in unrelated individuals | 203464 | -0.080 (-0.147, -0.013) | 0.019 |
| Degree | Women only | Siblings [Non-genetic, family effects] | 14346 | -0.033 (-0.086, 0.020) | 0.22 |
| Degree | Women only | Siblings [MR, family effects] | 14346 | -0.058 (-0.615, 0.499) | 0.84 |
| Have a skilled job | All | MR in unrelated individuals | 246844 | -0.144 (-0.224, -0.064) | 0.0004 |
| Have a skilled job | All | Siblings [Non-genetic, family effects] | 25609 | 0.007 (-0.048, 0.061) | 0.81 |
| Have a skilled job | All | Siblings [MR, family effects] | 25609 | 0.342 (-0.205, 0.890) | 0.22 |
| Have a skilled job | Men only | MR in unrelated individuals | 118302 | -0.182 (-0.314, -0.049) | 0.007 |
| Have a skilled job | Men only | Siblings [Non-genetic, family effects] | 5474 | 0.012 (-0.127, 0.151) | 0.86 |
| Have a skilled job | Men only | Siblings [MR, family effects] | 5474 | 0.160 (-1.17, 1.49) | 0.81 |
| Have a skilled job | Women only | MR in unrelated individuals | 128542 | -0.119 (-0.219, -0.019) | 0.02 |
| Have a skilled job | Women only | Siblings [Non-genetic, family effects] | 9081 | 0.002 (-0.081, 0.086) | 0.96 |
| Have a skilled job | Women only | Siblings [MR, family effects] | 9081 | -0.015 (-0.822, 0.792) | 0.97 |
| In employment | All | MR in unrelated individuals | 223821 | -0.039 (-0.232, 0.153) | 0.69 |
| In employment | All | Siblings [Non-genetic, family effects] | 23722 | -0.082 (-0.238, 0.075) | 0.31 |
| In employment | All | Siblings [MR, family effects] | 23722 | 0.593 (-0.757, 1.944) | 0.39 |
| In employment | Men only | MR in unrelated individuals | 109421 | -0.060 (-0.322, 0.201) | 0.65 |
| In employment | Men only | Siblings [Non-genetic, family effects] | 5114 | 0.110 (-0.213, 0.433) | 0.50 |
| In employment | Men only | Siblings [MR, family effects] | 5114 | 1.794 (-0.709, 4.297) | 0.16 |
| In employment | Women only | MR in unrelated individuals | 114400 | 0.007 (-0.296, 0.309) | 0.97 |
| In employment | Women only | Siblings [Non-genetic, family effects] | 8245 | -0.239 (-0.533, 0.055) | 0.11 |
| In employment | Women only | Siblings [MR, family effects] | 8245 | -0.214 (-2.435, 2.007) | 0.85 |
|  |  |  |  |  |  |
| Weekly visits from friends and family | All | MR in unrelated individuals | 377321 | -0.011 (-0.074, 0.051) | 0.72 |
| Weekly visits from friends and family | All | Siblings [Non-genetic, family effects] | 39477 | 0.049 (0.004, 0.093) | 0.034 |
| Weekly visits from friends and family | All | Siblings [MR, family effects] | 39477 | -0.208 (-0.664, 0.248) | 0.37 |
| Weekly visits from friends and family | Men only | MR in unrelated individuals | 173044 | -0.045 (-0.137, 0.048) | 0.34 |
| Weekly visits from friends and family | Men only | Siblings [Non-genetic, family effects] | 8032 | 0.095 (-0.007, 0.196) | 0.07 |
| Weekly visits from friends and family | Men only | Siblings [MR, family effects] | 8032 | 0.204 (-0.743, 1.151) | 0.67 |
| Weekly visits from friends and family | Women only | MR in unrelated individuals | 204277 | 0.018 (-0.068, 0.104) | 0.69 |
| Weekly visits from friends and family | Women only | Siblings [Non-genetic, family effects] | 14417 | 0.017 (-0.061, 0.096) | 0.67 |
| Weekly visits from friends and family | Women only | Siblings [MR, family effects] | 14417 | 0.532 (-0.279, 1.343) | 0.20 |
| Weekly participation in leisure and social activities | All | MR in unrelated individuals | 378615 | -0.057 (-0.112, -0.002) | 0.044 |
| Weekly participation in leisure and social activities | All | Siblings [Non-genetic, family effects] | 39561 | -0.053 (-0.090, -0.015) | 0.01 |
| Weekly participation in leisure and social activities | All | Siblings [MR, family effects] | 39561 | 0.063 (-0.313, 0.438) | 0.74 |
| Weekly participation in leisure and social activities | Men only | MR in unrelated individuals | 173796 | -0.076 (-0.164, 0.012) | 0.09 |
| Weekly participation in leisure and social activities | Men only | Siblings [Non-genetic, family effects] | 8065 | 0.006 (-0.087, 0.098) | 0.90 |
| Weekly participation in leisure and social activities | Men only | Siblings [MR, family effects] | 8065 | 0.486 (-0.397, 1.369) | 0.28 |
| Weekly participation in leisure and social activities | Women only | MR in unrelated individuals | 204819 | -0.044 (-0.115, 0.027) | 0.23 |
| Weekly participation in leisure and social activities | Women only | Siblings [Non-genetic, family effects] | 14426 | -0.098 (-0.157, -0.039) | 0.001 |
| Weekly participation in leisure and social activities | Women only | Siblings [MR, family effects] | 14426 | -0.012 (-0.594, 0.571) | 0.97 |
| Weekly confiding in close friend or relative | All | MR in unrelated individuals | 368555 | 0.010 (-0.049, 0.070) | 0.73 |
| Weekly confiding in close friend or relative | All | Siblings [Non-genetic, family effects] | 38511 | -0.019 (-0.059, 0.021) | 0.35 |
| Weekly confiding in close friend or relative | All | Siblings [MR, family effects] | 38511 | -0.120 (-0.514, 0.274) | 0.55 |
| Weekly confiding in close friend or relative | Men only | MR in unrelated individuals | 168649 | 0.026 (-0.064, 0.116) | 0.58 |
| Weekly confiding in close friend or relative | Men only | Siblings [Non-genetic, family effects] | 7796 | 0.033 (-0.06, 0.127) | 0.49 |
| Weekly confiding in close friend or relative | Men only | Siblings [MR, family effects] | 7796 | -1.068 (-1.93, -0.211) | 0.015 |
| Weekly confiding in close friend or relative | Women only | MR in unrelated individuals | 199906 | -0.001 (-0.081, 0.079) | 0.98 |
| Weekly confiding in close friend or relative | Women only | Siblings [Non-genetic, family effects] | 14092 | -0.021 (-0.087, 0.046) | 0.54 |
| Weekly confiding in close friend or relative | Women only | Siblings [MR, family effects] | 14092 | 0.098 (-0.559, 0.756) | 0.77 |
| Cohabitation with partner or spouse | All | MR in unrelated individuals | 348570 | -0.067 (-0.135, 0.002) | 0.06 |
| Cohabitation with partner or spouse | All | Siblings [Non-genetic, family effects] | 35735 | -0.062 (-0.108, -0.017) | 0.01 |
| Cohabitation with partner or spouse | All | Siblings [MR, family effects] | 35735 | -0.049 (-0.480, 0.382) | 0.82 |
| Cohabitation with partner or spouse | Men only | MR in unrelated individuals | 163828 | 0.094 (-0.017, 0.205) | 0.10 |
| Cohabitation with partner or spouse | Men only | Siblings [Non-genetic, family effects] | 7460 | 0.088 (-0.031, 0.207) | 0.15 |
| Cohabitation with partner or spouse | Men only | Siblings [MR, family effects] | 7460 | 0.077 (0.002, 0.152) | 0.045 |
| Cohabitation with partner or spouse | Women only | MR in unrelated individuals | 184742 | -0.173 (-0.261, -0.086) | 0.00009 |
| Cohabitation with partner or spouse | Women only | Siblings [Non-genetic, family effects] | 12705 | -0.094 (-0.165, -0.023) | 0.009 |
| Cohabitation with partner or spouse | Women only | Siblings [MR, family effects] | 12705 | -0.628 (-1.288, 0.032) | 0.06 |
